# *Langbiangia*, a new genus of Gesneriaceae endemic to Langbiang Plateau, southern Vietnam and a taxonomic endeavor to achieve key targets of the post-2020 global biodiversity framework

**DOI:** 10.1371/journal.pone.0284650

**Published:** 2023-05-17

**Authors:** Hong Truong Luu, Chia-Lun Hsieh, Chia-Rong Chuang, Cheng-Wei Chen, Ngoc Toan Tran, Ngoc Long Vu, Kuo-Fang Chung

**Affiliations:** 1 Southern Institute of Ecology, Institute of Applied Materials Science & Graduate University of Science and Technology, Vietnam Academy of Science and Technology, Ho Chi Minh City, Vietnam; 2 Research Museum and Herbarium (HAST), Biodiversity Research Center, Academia Sinica, Taipei, Taiwan; 3 Biodiversity Program, Taiwan International Graduate Program, Academia Sinica, Taipei, Taiwan; 4 Department of Life Science, National Taiwan Normal University, Taipei, Taiwan; Bangladesh Agricultural University, BANGLADESH

## Abstract

Situated in the southern end of the Annamite Mountain Range, Langbiang Plateau is a major biodiversity hotspot of southern Vietnam known for high species diversity and endemicity. To achieve effective conservation, parts of the plateau were designated as the Langbiang Biosphere Reserve, an UNESCO World Network aiming to improve relationships between inhabitants and their environments. Amongst the rich endemic flora of the plateau are three gesneriads ascribed to *Primulina*, a calciphilous genus with high species diversity in the vast limestone karsts stretching from southern China to northern Vietnam. However, a recent phylogenetic study questioned the generic placement of the Langbiang *Primulina*, corroborating with observations on the geographical distribution, habitat preference, and phyllotaxy of the three species. Based on phylogenetic analyses of nuclear ITS and plastid *trnL-F* DNA sequences of a comprehensive sampling covering nearly all genera of the Old World Gesneriaceae, we demonstrate that the three Langbiang *Primulina* species form a fully supported clade distantly related to other *Primulina*. As this clade is biogeographically, ecologically, morphologically, and phylogenetically distinct worthy of generic recognition, we propose to name it ***Langbiangia* gen. nov.** to highlight the rich and unique biodiversity of the Langbiang Plateau. By means of this taxonomic endeavor, we are hoping to raise the conservation awareness of this biodiversity heritage of southern Vietnam and promote the importance of Langbiang Biosphere Reserve that is crucial for achieving action-oriented global targets of the post-2020 global biodiversity framework (GBF) of the UN Convention on Biological Diversity (CBD)—effective conservation and management of at least 30% of biodiverse terrestrial, inland water, and costal and marine areas by 2030—that has been agreed at the COP15 in Montréal in December 2022.

## Introduction

**Fig 1:**
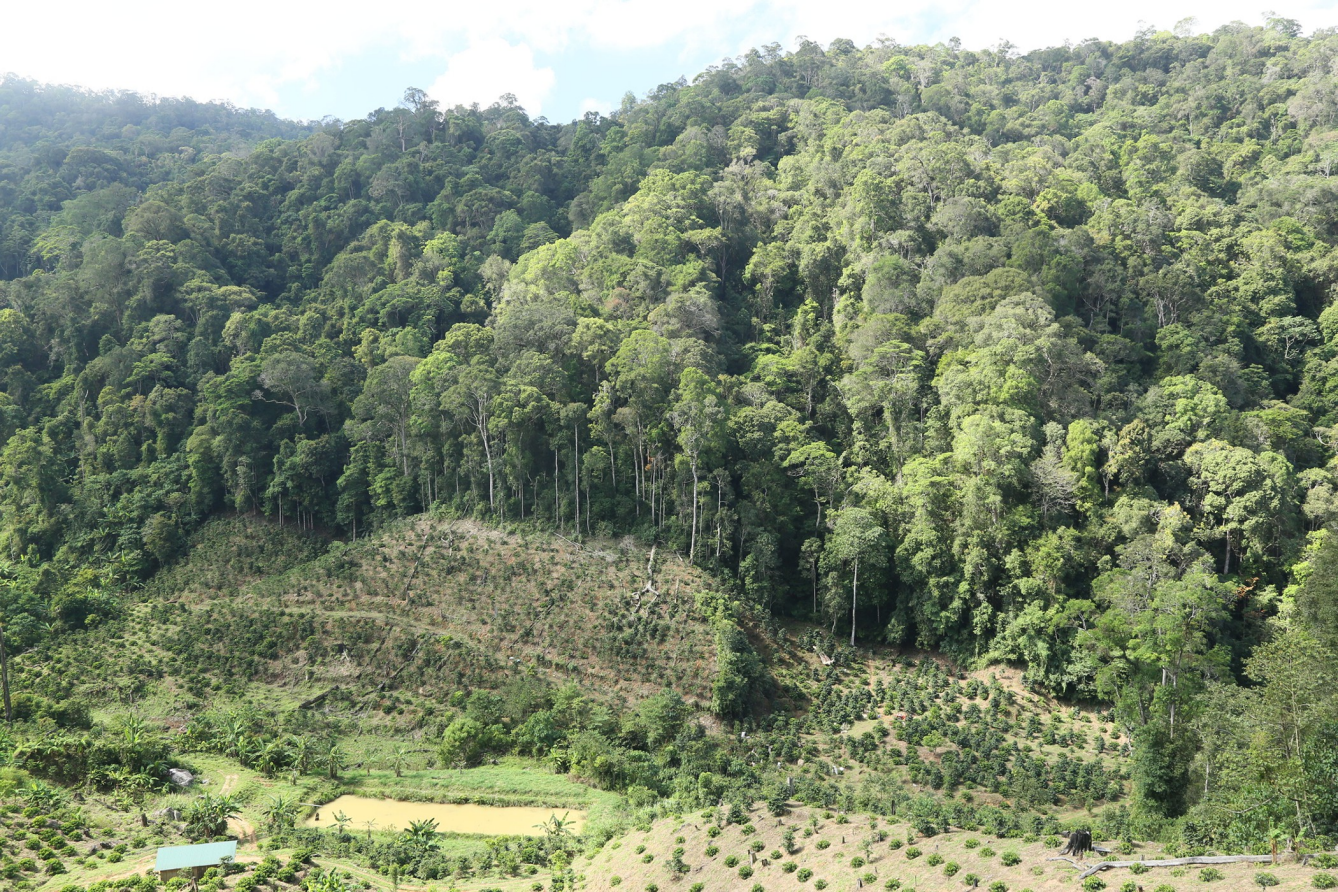
Broad-leaved evergreen forest and expanding coffee plantation in Langbiang Plateau. As in much of the tropical countries, the biodiverse montane forests of the Langbiang Plateau are being cleared for agriculture such as coffee plantation, threatening the native flora and fauna and the ecosystem functions (photo: C.-W. Chen).

The realization that collaboration with environmental and natural resource stakeholders (Fig 1) is essential for effective conservation of biodiversity [1, 2] has instigated the ideas of “other effective area-based conservation measures (OECMs)” to complement traditional protected and preserved areas such as national parks and nature reserves [3]. An example of the OECMs is UNESCO (United Nations Educational, Scientific and Cultural Organization) Biosphere Reserves (BRs) [4]. First introduced in 1971, UNESCO Biosphere Reserves are a global network of “*learning places for sustainable development*” using “*interdisciplinary approaches to understanding and managing changes and interactions between social and ecological systems*, *including conflict prevention and management of biodiversity*” [5]. As of now, the World Network of Biosphere Reserves consists of 738 sites in 134 countries aiming to improve relationships between inhabitants and their environments [5]. Given their integrated nature, UNESCO BRs can facilitate greatly the achievement of the action-oriented global targets of the post-2020 global biodiversity framework (GBF) agreed at the 15^th^ meeting of the Conferences of Parties (COP 15) to the UN Convention on Biological Diversity (CBD) in Montréal in December 2022 [6]—effective conservation and management of at least 30% of biodiverse terrestrial, inland water, and costal and marine areas by 2030 [4]. Unfortunately, although current UNESCO BRs cover more than 7 million km^2^ and could contribute ca. 4.5% to the conventional area-based protected areas [4], Biosphere Reserves are in general far less recognized than traditional protected areas such as national parks [7]. The issue is particularly dire in developing countries such as Vietnam, which boasts a remarkably high biodiversity [8, 9] and is home to 11 UNESCO Biosphere Reserves, the second-highest number in SE Asia [10–12]. To fully implement functions of UNESCO BRs, the importance of awareness-raising and outreach has constantly been emphasized [7, 12, 13].

**Fig 2:**
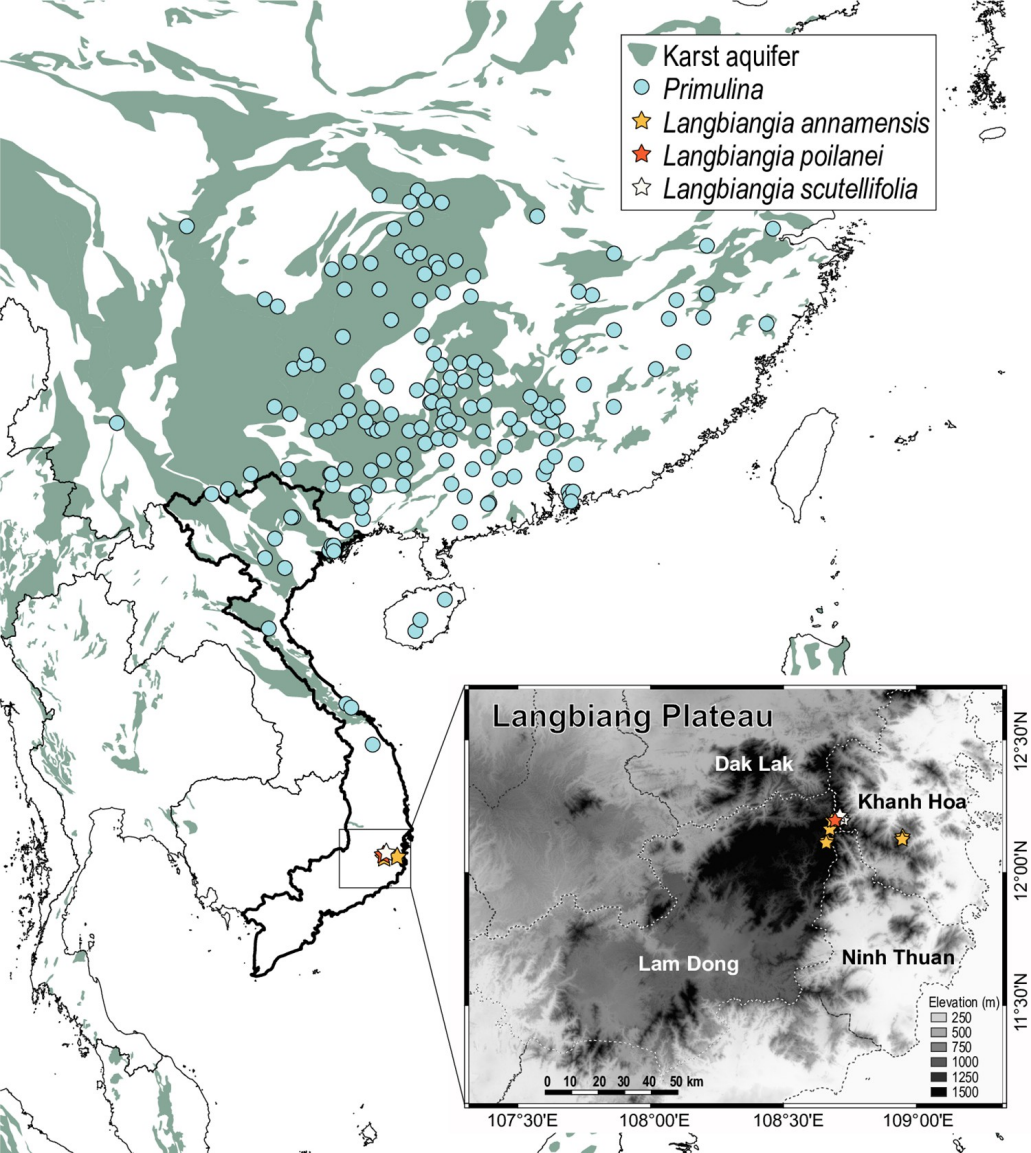
Distribution of *Langbiangia* and *Primulina*. The map was generated using QGIS 3.24.1 [60]. The basemap of karst aquifers was downloaded from WHYMAP [61], which can be freely used and copied for educational and other non-commercial purposes. The distributions were mapped based on occurrence records retrieved from Global Biodiversity Facility [62]. The distribution of *Langbiangia poilanei* was plotted based on information taken from the holotype. This work is licensed under a CC BY 4.0 license.

Located within the Langbiang Plateau (Figs 1 and 2), Langbiang Biosphere Reserve is the 9^th^ UNESCO BRs in Vietnam [14]. Langbiang Plateau (also spelled as Lam Vien Plateau or known as Da Lat Plateau) is situated in the southern end of the Annamite Mountain Range and bordered by the Provinces of Dak Lak, Khanh Hoa, Lam Dong, and Ninh Thuan of Vietnam (Fig 2). As a long-recognized biodiversity hotspot of southern Vietnam, a portion of the Langbiang Plateau covering 275,439 ha has been protected as the Bidoup—Nui Ba National Park, which is also the core zone of the Langbiang Biosphere Reserve designated by UNESCO in 2015 [14]. Langbiang Biosphere Reserve is home to many rare and endangered mammals such as Indochinese tiger, yellow-cheeked gibbon, Indian bison, etc. [14]. Additionally, a rich diversity in bats has also been documented in the region [15]. In the recent decade, several new vertebrates including six fishes [16, 17], three snakes [18–20], and four amphibians [21–24] were described from the Langbiang Plateau. Across the plateau, cryptic genetic diversity was also detected in the frog genus *Leptolalax* [25]. The plateau is also known for its high fungal diversity [26, 27] and the only habitat of the rare holoparasitic plant *Sapria himalayana* Griff. (Rafflesiaceae) in Vietnam [28]. Langbiang Plateau is also known as the prime habitats of numerous vascular plant species and in the recent decade, a wide range of vascular plant taxa have also been described and/or recorded, including 16 ferns [29–32], one Schisandraceae [33], three Lauraceae [33, 34], five orchids [35, 36], one bamboo [37], one Zingiberaceae [33], one legume [38], one aroid [39], one Menispermaceae [40], one Daphniphyllaceae [33], four stone oaks [41, 42], one Euphorbiaceae [43], two Rosaceae [33], two begonias [44], one Clusiaceae [45], one Rutaceae [33], seven camellias [46–50], four Nyssaceae [33, 51], one ginseng [52], one Loranthaceae [53], two hollies [33], one Icacinaceae [33], one Symplocaceae [33], one Rubiaceae [54], and five gesneriads [55–58]. However, the rich and unique biological diversity of the Langbiang Plateau has continuously been threatened by growing land demands for agriculture (Fig 1) and residency [59]. These land-use changes thus underline the function of UNESCO World Network of Biosphere Reserves for sustainable development of the biodiverse Langbiang Plateau.

The predominantly Old World subfamily Didymocarpoideae of the African Violet Family Gesneriaceae is composed of ca. 2,500 species [63]. In recent decades, recorded diversity in the subfamily has grown considerably as new taxa are being described continuously [e.g., 58, 64–66] especially in Vietnam [55–58, 66–71]. However, molecular phylogenetic studies in the past decade also showed that traditional taxonomy of Gesneriaceae had utilized traits that are homoplasious [72–76]. Consequently, infrafamilial classification and generic delimitation of Gesneriaceae have undergone drastic changes in the past two decades [63, 77]. One of the most extreme examples of such changes has been *Chirita* Buch.-Ham. ex D.Don. Traditionally, *Chirita* was known by the possession of a bifid stigma characterized by undeveloped upper lobe and bifidly developed lower lobe known as the chiritoid stigma [75, 78]. However, molecular phylogenetic analyses showed that *Chirita* was highly polyphyletic [75, 79, 80], with the type species *C*. *urticifolia* Buch.-Ham. ex D.Don intermingled with *Henckelia* Spreng. that was published earlier than *Chirita* and thus has the taxonomic priority [75]. To establish a stable and phylogeny-based taxonomy of Gesneriaceae, the name *Chirita* was synonymized and its constituted species were transferred to *Codonoboea* Ridl., *Damrongia* Kerr ex Craib, *Henckelia*, *Microchirita* (C.B.Clarke) Yin Z.Wang (≡*Chirita* sect. *Microchirita* C.B.Clarke), *Liebigia* Endl. [≡*Chirita* sect. *Liebigia* (Endl.) C.B.Clarke], and *Primulina* Hance (≡*Chirita* sect. *Gibbosaccus* C.B.Clarke) [75].

With the inclusion of former members of *Chirita* sect. *Gibbosaccus* as well as *Chiritopsis* W.T.Wang and *Wentsaiboea* D.Fang & D.H.Qin [64, 80], and the addition of more than 70 new species, the recircumscribed *Primulina* has been expanded from a monotypic genus to its current size of 227 species [64, 81, 82]. However, subsequent phylogenetic analyses revealed that some former members placed in *Chirita* sect. *Gibbosaccus* by Wood 1974 [78] and transferred to *Primulina* without DNA data by Weber et al. 2011 [75] should be placed in *Deinostigma* W.T.Wang & Z.Y.Li [83, 84]. More recently, *Chirita umbrophila* C.Y.Wu ex H.W.Li, a species with uncertain generic placement [75], was shown to be conspecific with *Loxostigma kurzii* (C.B.Clarke) B.L.Burtt [85]. On the other hand, phylogenetic studies of materials sampled from further botanical exploration also revealed lineages that are distinct and worthy of generic recognition (e.g., *Actinostephanus* F.Wen, Y.G.Wei & L.F.Fu [65], *Billolivia* D.J.Middleton [86], *Chayamaritia* D.J.Middleton & Mich.Möller [87], *Glabrella* Mich.Möller & W.H.Chen [88], *Michaelmoelleria* F.Wen, Y.G.Wei & T.V.Do [66], *Middletonia* C.Puglisi [89], *Rachunia* D.J.Middleton & C.Puglisi [90], etc.). These recent taxonomic changes and novelties of the didymocarpoid Gesneriaceae underscore the importance of incorporating molecular information in achieving a stable classification of Asian Gesneriaceae.

**Fig 3:**
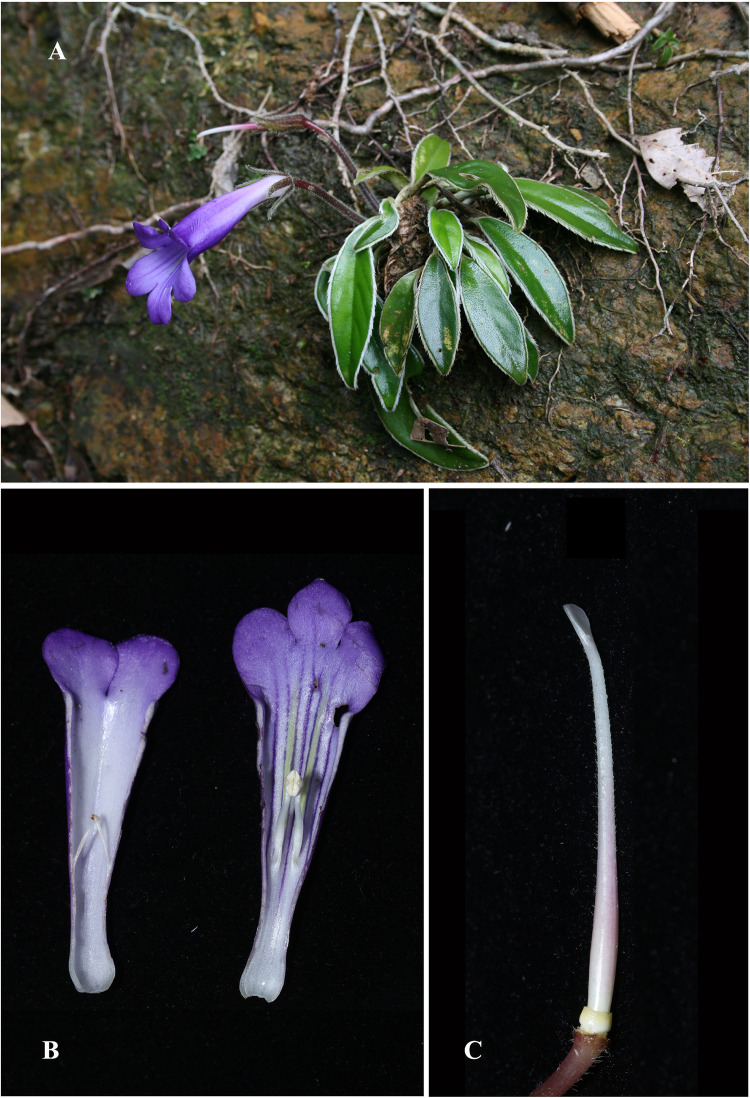
*Langbiangia poilanei*. (A) Habit. (B) Corolla, longitudinal dissection with stamens. (C) Disc, ovary and style. Photos by Hong Truong Luu and Minh Tri Dang.

Among the five recently described gesneriads from the Langbiang Plateau, *Primulina scutellifolia* Luu, N.L.Vu & T.Q.T.Nguyen [58] is the latest described species. On the Langbiang Plateau, *P*. *scutellifolia* is distributed in close proximity with *P*. *poilanei* (Pellegr.) Mich.Möller & A.Weber (Fig 3) and often sympatric with *P*. *annamensis* (Pellegr.) Mich.Möller & A.Weber [58] (Fig 2). Both *P*. *annamensis* and *P*. *poilanei* were initially described as *Chirita* Buch.-Ham. ex D. Don (i.e., *C*. *poilanei* Pellegr. [91] and *C*. *annamensis* Pellegr. [92, 93]) and placed under *Chirita* sect. *Gibbosaccus* by Wood 1974 [78]. Along with other species of *Chirita* sect. *Gibbosaccus*, *C*. *annamensis* and *C*. *poilanei* were both transferred to *Primulina* in Weber et al. 2011 [75], though neither of these two species were sampled for phylogenetic analyses. *Primulina scutellifolia* was described as a morphologically similar species of *P*. *annamensis*, differing from the latter by several morphological characteristics including scutellate leaves, less densely pilose petioles, and the stigma with enlarged lower lip but without upper lip [58]. Likewise, no molecular data was generated for *P*. *scutellifolia* to assure its generic placement [58].

**Fig 4:**
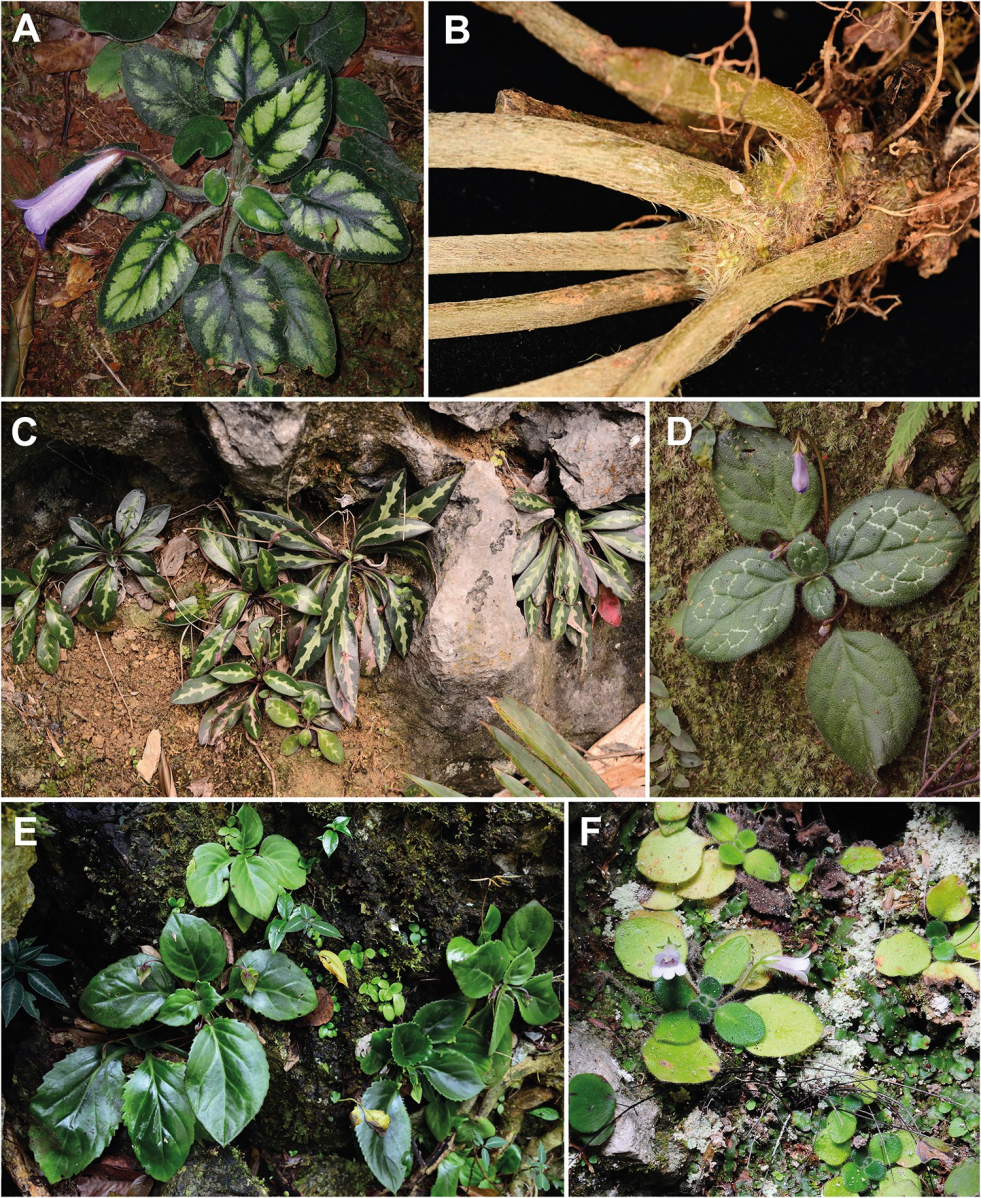
Phyllotaxy of *Langbiangia* and *Primulina*. (A) *Langbiangia annamensis*, photo by Maxim S. Nuraliev / CC BY-NC 4.0 (https://www.inaturalist.org/observations/38536524) (B) The acaulescent stem of *Langbiangia scutellifolia*. (C) *Primulina minutimaculata*. (D) *Primulina dryas*. (E) *Primulina lungzhouensis*. (F) *Primulina minor*. B–E by C.-L. Hsieh, and F by K.-F. Chung.

Biogeographically, *Primulina annamensis*, *P*. *poilanei*, and *P*. *scutellifolia* are all restricted to the Langbiang Plateau, representing the southernmost and disjunctively distributed species of *Primulina* (Fig 2). Ecologically, while a majority of *Primulina* are calciphilous plants on limestone karsts (Fig 2) of lower elevations [64, 94–96], *P*. *annamensis*, *P*. *poilanei*, and *P*. *scutellifolia* grow on humid fertile soils of the evergreen broad-leaved forest at elevation of 1,450–2,000 m [58, 78, 92, 93, 97]. Morphologically, *P*. *annamensis*, *P*. *poilanei*, and *P*. *scutellifolia* also differ from the typical *Primulina* by their alternate and spirally arranged phyllotaxy [58, 78], while a majority of *Primulina* are characterized by decussate or whorled leaves (Fig 4). Additionally, the peltate and scutellate leaf bases of *P*. *scutellifolia* are reminiscent to *Deinostigma poilanei* (Pellegr.) W.T.Wang & Z.Y.Li [98], *D*. *serratum* F.Wen, L.N.Tuan & D.Dien [99], and *D*. *tamianum* (B.L.Burtt) D.J.Middleton & H.J.Atkins [58] not known in any other species of *Primulina*. Prior to its current placement in *Deinostigma* W.T.Wang & Z.Y.Li, *D*. *tamianum* [83] was also first described as *Chirita* (i.e., *Chirita tamiana* B.L.Burtt) and classified under *Chirita* sect. *Gibbosaccus* [78]. Along with *C*. *annamensis* and *C*. *poilanei*, *C*. *tamiana* was also transferred to *Primulina* without molecular data [75], and was later transferred to the expanded *Deinostigma* [84]. Morphologically, however, in contrast to the caulescent habit and bilocular ovaries observed in *Deinostigma*, all three Langbiang *Primulina* are acaulescent with unilocular ovaries. Phylogenetically, Xu et al. 2021 [95] showed that their sampled *Primulina annamensis* (i.e., *Primulina annamensis* ANNA) was not placed within the ‘true’ *Primulina* but sister to their sampled *Deinostigma poilanei* (i.e., *Deinostigma poilanei* POIL), though no other *Deinostigma* species were sampled and the taxonomic implications were not discussed. Therefore, it remains uncertain whether the three Langbiang *Primulina* species belong to *Primulina*, *Deinostigma*, or even a unique genus of itself. The biogeographical, ecological (Fig 2), morphological (Figs 3 and 4), and phylogenetic [95] uniqueness of the Langbiang Plateau *Primulina* thus prompted us to conduct phylogenetic analyses to confirm generic placements of the three species.

## Materials and methods

### Ethics statement

The samples used in this studied were collected under the permits granted by Van Huong Le, the director of Bidoup—Nui Ba National Park.

### Inclusivity in global research

Additional information regarding the ethical, cultural, and scientific considerations specific to inclusivity in global research is included in the S1 Checklist.

### Sampling and DNA extraction

To test generic placements of the three Langbiang *Primulina* species, we sampled two individuals of *P*. *annamensis*, two *P*. *poilanei*, and one *P*. *scutellifolia*. The material of *P*. *scutellifolia*, which was assessed as Critically Endangered [58], was sampled from the type collection. As *P*. *scutellifolia* was considered morphologically similar to *Deinostigma* [58], we also collected *D*. *eberhardtii* (Pellegr.) D.J.Middleton & H.J.Atkins (Bach 0512), which previously has never been analyzed molecularly, to complement the sampling of the genus. Additionally, Bach 0513, which is an unknown gesneriad akin to *Deinostigma*, was also added to the analyses. Voucher specimens (Table 1) were deposited in the Herbarium of Southern Institute of Ecology (SGN), Ho Chi Minh City, Vietnam. The genomic DNAs were extracted from 0.2 g of silica-dried leaf material using the CTAB method [100]. The quantity and quality of the extracted DNAs were checked by electrophoresis on 1.5% agarose gels.

**Table 1:** Sampling information and GenBank accession numbers of newly sequenced individuals. All voucher specimens were deposited in the Herbarium of Southern Institute of Ecology (SGN).

Code	Species	Voucher	Locality	GenBank accession numbers (*trnL-F*/ITS)
Bach 0512	*Deinostigma eberhardtii*	*Tran Ngoc Toan Bach 0512*	Da Nang City, Lien Chieu District, Nam Hai Van Special Use Forest	OP617258/OP605498
Bach 0513	*Deinostigma sp*.	*Tran Ngoc Toan Bach 0513* (living collection, SGN)	Quang Nam Province, Nong Son District, Quang Nam Elephant Sanctuary	OP617259/OP605499
BD77	*Primulina annamensis*	*Dang Minh Tri BD-TN3-077*	Lam Dong Province, Lac Duong District, Da Chais Commune, Bidoup—Nui Ba National Park, Bidoup Forest Dynamics Plot	OP508008/OP508034
CR024	*Primulina annamensis*	*C*.*-W*. *Chen s*.*n*.	Lam Dong Province, Lac Duong District, Giang Ly Ranger Station	OP617260/OP605500
CR027	*Primulina poilanei*	*C*.*-W*. *Chen s*.*n*.	Khanh Hoa Province, Khanh Vinh District	OP617261/OP605501
CR028	*Primulina poilanei*	*C*.*-W*. *Chen s*.*n*.	Khanh Hoa Province, Khanh Vinh District	OP617262/OP605502
Luu KH0945	*Primulina scutellifolia*	*Luu KH0945*	Khanh Hoa Province, Khanh Vinh District, Son Thai Commune	OP508007/OP508033

Based on the most updated classification of Gesneriaceae, *Primulina* and *Deinostigma* are both placed in subtribe Didymocarpinae, tribe Trichosporeae, subfamily Didymocarpoideae [63, 77, 96, 101]. To maximize taxon sampling while exploiting DNA sequences available in the GenBank, we sequenced the internal transcribed spacer (ITS) of the nuclear ribosomal DNA (including the 5.8S gene) and the plastid *trnL-trnF* intergenic spacer (*trnL-F*), which have been the most widely used molecular markers in reconstructing phylogenetic relationships of Asian Gesneriaceae and delimitating new gesneriad genera [74–76, 86–88, 90, 102, 103]. The ITS sequence was amplified by the universal primers, ITS4 and ITS5 [104]. The *trnL-F* was amplified using three primer pairs: trnL-F_1F (5ʹ-GGCGAAATCGGTAGACGCTA-3ʹ) and trnL-F_1R (5ʹ-CCCAGATACAGATTCGGGCC-3ʹ), trnL-F_2F (5ʹ-GGCCCGAATCTGTATCTGGG-3ʹ) and trnL-F_2R (5ʹ-ACCGTTAACGAACAAAGCGG-3ʹ), and trnL-F_3F (5ʹ-CCGCTTTGTTCGTTAACGGT-3ʹ) and trnL-F_3R (5ʹ-CCATGTGCCAGGAACCAGAT-3ʹ), designed using Primer3 v.2.3.7 [105] implemented in the program Geneious Prime® v.2022.1.1 [106]. The total volume for all polymerase chain reactions (PCRs) was 30 μl with 15 μl of Q-AmpTM 2x ScreeningFire Taq Master Mix (Bio-Genesis Technologies Inc., Taiwan), 9 μl of ddH2O, 4 μl of DNA, and 1 μl of each of the two primers. All PCRs were carried out under the thermocycling program which starts from 5 minutes at 95° C for initial denaturation, followed by 40 cycles of amplification of 1 minute at 95° C, 1 minute at 56° C for, and 1 minute at 72° C, and ends with the final extension step of 7 minutes at 72° C. The PCR products were Sanger sequenced by Genomics BioScience and Technology Co. (Taipei, Taiwan). All newly generated DNA sequences (S1 Table) have been deposited in GenBank (https://www.ncbi.nlm.nih.gov/genbank/).

### Sequence alignment and phylogenetic analyses

Geneious was used to conduct sequencing quality check and sequence assembly. In addition to the newly generated sequences, DNA sequences of 256 species, representing nearly all genera, all subtribes, and all tribes (i.e., Epithemateae and Trichosporeae) of subfamily Didymocarpoideae, were downloaded from GenBank (S1 Table). Our sampling of the subtribe Didymocarpinae included 135 species representing all genera except for the doubtful genus *Sepikea* Schltr. [63]. To ease the computational loading, we only sampled 16 species of *Primulina* that represent all major clades of the genus [64]. *Titanotrichum oldhamii* (Hemsl.) Soler. of tribe Titanotricheae, subfamily Gesnerioideae was chosen to root the tree based on recent studies [63, 77]. After incorporating the newly generated sequences, each of the ITS and *trnL-F* matrix was aligned separately by MAFFT v.7.490 [107] using Geneious with default settings. Each sequence in the alignment was manually trimmed to the same length, and the sites with >98% of gaps were excluded by applying the “mask alignment” function in Geneious.

Because of low phylogenetic resolutions in preliminary maximum likelihood (ML) analyses of individual matrices (S1 and S2 Figs) using RAxML v.8.2.11 [108], the two matrices were concatenated (S1 File) as executed in a majority of Gesneriaceae phylogenetic analyses [74–76, 86, 87, 90, 102, 103] using AMAS python scripts [109]. For the concatenated matrix, we conducted phylogenetic analyses based on both (ML) and Bayesian inference (BI) using RAxML v.8.2.11 and MrBayes v.3.2.7a [110], respectively. ML analyses were conducted with the settings of GTR+GAMMA for nucleotide substitution model and rapid bootstrap (-f a option) with 2000 replicates. Nodes in the ML tree with bootstrap support (BS) lower than 50% were collapsed into polytomies by using TreeCollapserCL 4 [111]. Prior to the BI analyses, the best-fit nucleotide model for each partition was determined by ModelFinder [112] in IQ-TREE v.2.0.6 [113] with the setting of -mset mrbayes. For BI analyses, according to ModelFinder’s results, the SYM+I+G and GTR+F+G models were applied to ITS and *trnL-F* partitions, respectively, in MrBayes with all model parameters unlinked among partitions and allowing the overall rate to vary across partitions. In MrBayes, two independent runs with four chains each of the Markov Chain Monte Carlo (MCMC) simulations were set to perform 10,000,000 generations with sampling frequency of every 1000 generations and the first 25% of generations discarded as burn in. The resulting trees were summarized into a phylogram with clade credibility (posterior probability) values. The final phylogenies of ML and BI analyses were visualized in FigTree v.1.4.4 [114].

### Nomenclature

The electronic version of this article in Portable Document Format (PDF) in a work with an ISSN or ISSBN will represent a published work according to the International Code of Nomenclature for algae, fungi, and plants, and hence the new names contained in the electronic publication of a *PLOS ONE* article are effectively published under that Code from the electronic edition alone, so there is no longer any need to provide printed copies.

In addition, new names contained in this work have been submitted to IPNI, from where they will be made available to the Global Names Index. The IPNI LSIDs can be resolved and the associated information viewed through any standard web browser by appending the LSID contained in this publication to the prefix http://ipni.org/. The online version of this work is archived and available from the following digital repositories: PubMED Central and LOCKSS.

## Results and discussion

**Fig 5:**
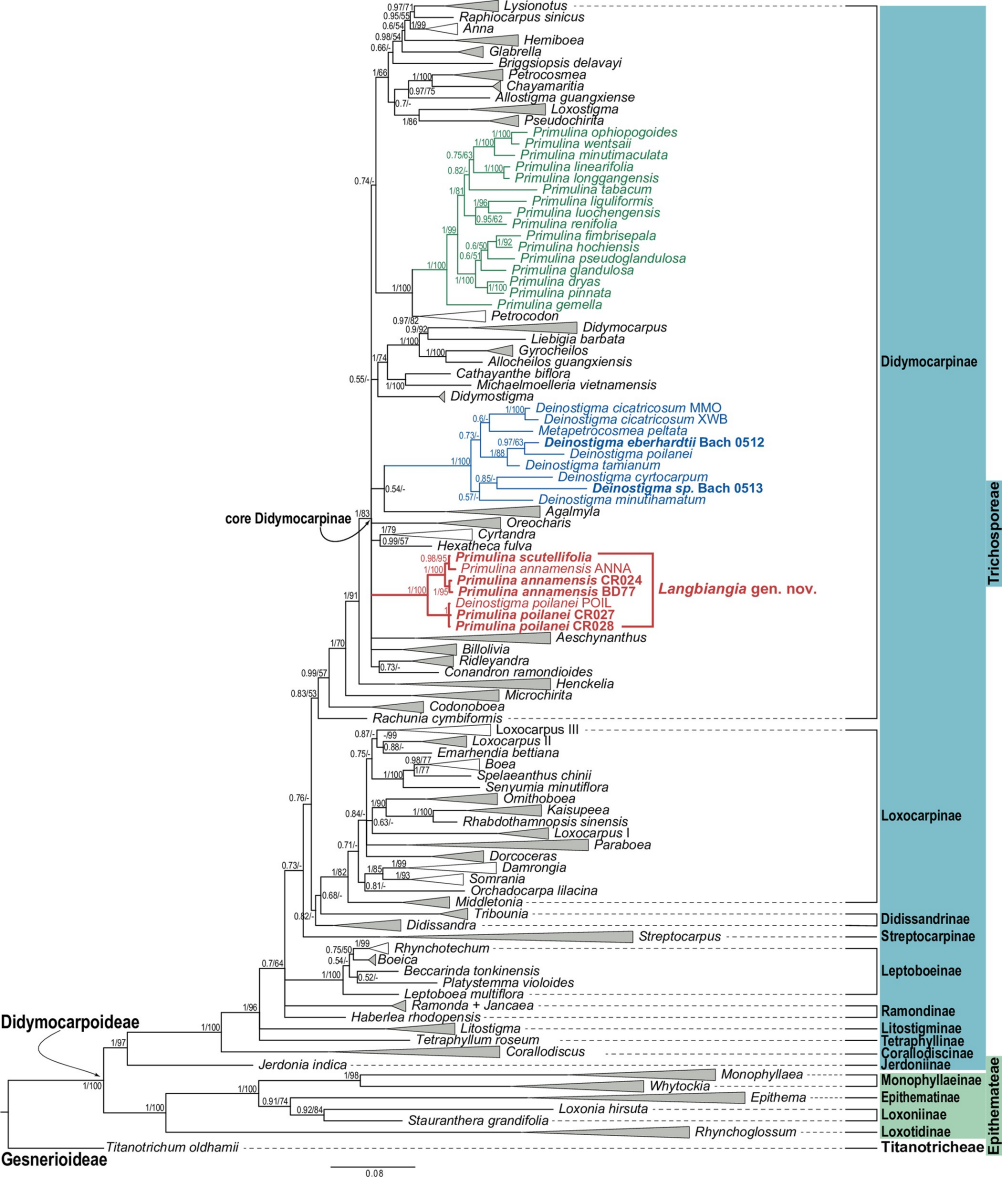
Simplified Bayesian inference tree of Didymocarpoideae based on combined *trnL-F* and ITS sequences. Numbers at the node indicate Bayesian posterior probability (≥ 0.5), with ML bootstrap value (≥ 50%) shown after slash (/). All genera except for *Primulina*, *Deinostigma* and *Langbiangia* **gen. nov.** are collapsed into triangles, with gray triangles denoting genera/clades that are fully supported in both Bayesian and ML analyses. The infrafamilial classification of Weber et al. 2013 [77] and Weber et al. 2020 [63] is followed. This tree is simplified from the S3 Fig. ML bootstrap values are retrieved from S5 Fig.

The DNA alignment of 242 ITS sequences was 956 bp in length with 838 (87.7%) parsimony informative sites, while the *trnL-F* alignment of 252 sequences was 931 bp in length with 604 (64.9%) parsimony informative sites. The final concatenated matrix of 256 accessions was 1,887 bp in length with 1,136 (60.2%) parsimony informative sites. Results of BI (Fig 5 and S3 Fig) and ML (S4 and S5 Figs) analyses are largely congruent, with BI analysis showing better resolution and higher support values (S3 and S5 Figs). A simplified Bayesian inference phylogeny with ML bootstrap support values is shown in Fig 5. Rooted by *Titanotrichum oldhamii* of the subfamily Gesnerioideae, the monophyly of both tribes Epithemateae and Trichosporeae of the subfamily Didymocarpoideae is recovered (Fig 5). Within the tribe Trichosporeae, subtribes Jerdoniinae, Corallodiscinae, Tetraphyllinae, and Litostigminae formed successive sister clades to the rest of the subtribes as revealed in previous studies [63, 77]. Relationships among the rest of the subtribes, i.e., Ramondinae, Leptoboeniae, Streptocarpinae, Didissandrinae, Loxocarpinae, and Didymorcarpinae, remained unresolved or poorly resolved (Fig 5).

Within the subtribe Didymocarpinae, *Rachunia*, *Codonoboea*, *Microchirita*, and *Henckelia* formed successive sister groups to an unresolved clade composed of all other genera of the subtribe (i.e., ‘core’ Didymocarpinae), congruent with previous studies [66, 90]. As shown in previous phylogenetic studies of Didymocarpinae using ITS and *trnL-F* sequences, relationships within the ‘core’ Didymocarpinae remained poorly resolved [75, 77, 79, 86, 87, 90]. The employment of genomic data such as whole plastome sequences [96] and target capture [101] will be essential to clarify and understand this apparent radiation of Asian gesneriad clade.

Together with *Primulina annamensis* ANNA and *Deinostigma poilanei* POIL that were sequenced by Xu et al. 2021 [95], our newly sequenced Langbiang *Primulina* formed a fully supported clade within the ‘core’ Didymocarpinae (Fig 5), while other sampled species of ‘true’ *Primulina* formed a fully supported clade sister to *Petrocodon* Hance as revealed in previous studies [64, 96]. Except for *D*. *poilanei* POIL [95], all other sampled *Deinostigma* and its sister genus *Metapetrocosmea*, including our newly sequenced *D*. *eberhardtii* Bach 0512 and *Deinostigma sp*. Bach 0513, as well as *D*. *poilanei* KN201 that was used for recircumscribing *Deinostigma* by Möller et al. 2016 [83], formed a fully support clade (Fig 5). Although no detailed voucher information was provided for *D*. *poilanei* POIL [95], given that both ITS (S1 Fig) and *trnL-F* (S2 Fig) sequences of *D*. *poilanei* POIL are almost identical to our sequenced Langbiang *Primulina*, our phylogenetic results suggest that *D*. *poilanei* POIL could be a confusion with and/or misidentification of *Primulina poilanei*, likely resulted from the identical species epithet ‘poilanei’.

Within the Langbiang *Primulina* clade, *P*. *annamensis* ANNA sequenced by Xu et al. 2021 [95] is sister to *P*. *scutellifolia* collected from the type collection in both the ITS tree (S1 Fig) and the concatenated tree (Fig 5), rendering *P*. *annamensis* paraphyletic, though relationships among the three Langbiang *Primulina* species are unresolved in the *trnL-F* tree (S2 Fig). Because *Primulina scutellifolia* is morphologically similar to *P*. *annamensis* [58], *P*. *annamensis* ANNA sampled by Xu et al. 2021 [95] might also be a misidentification of *P*. *scutellifolia*. Alternatively, given the geographical proximity between *P*. *annamensis* and *P*. *scutellifolia* [58], the paraphyly of *P*. *annamensis* with *P*. *scutellifolia* nested within (Fig 5) also suggests the possibility of hybridization between the two species. Nevertheless, further study with increase sampling and additional molecular markers will be needed to clarify the species boundaries between these two species.

Given the biogeographical, ecological (Fig 2), morphological (Figs 3 and 4), and phylogenetic distinctness (Fig 5) of the Langbiang *Primulina*, our study clearly shows that the clade composed of these three species should be recognized as a distinct genus as those recently described genera of the subtribe Didymocarpinae [65, 66, 86–90]. With the reduction of these three species, the total number of *Primulina* known from Vietnam decreases from 24 [58] to 21 species. As members of this new genus are thus far only known from the Langbiang Plateau, we propose to name the new genus *Langbiangia* to highlight the rich and unique biodiversity of the region. In naming the new genus *Langbiangia*, we are also hoping to raise the conservation awareness and popularize the importance of Langbiang Biosphere Reserves that is crucial for achieving action-oriented global targets of the post-2020 GBF of the UN CBD [6].

### Taxonomic treatment

***Langbiangia*** Luu, C.L.Hsieh & K.F.Chung, **gen. nov.** [urn:lsid:ipni.org:names:77306595–1]

Type: *Langbiangia poilanei* (Pellegr.) Luu, C.L.Hsieh & K.F.Chung

**Diagnosis.** *ngbiangia* shares many similar floral traits with *Primulina*, but differs from the latter by its spirally-arranged alternate phyllotaxy. *Langbiangia* is also reminiscent of *Deinostigma*, but could be distinguished by the acaulescent habit and unilocular ovary in *Langbiangia*.

**Description.** aulescent herbaceous perennials. *Rhizomes* terete, sometimes woody, vertical or horizontal, 3–7 mm in diameter, up to 9 cm long. *Leaves* leathery, 3–13, spirally alternate, rosulate; petioles rounded or cylindrical, 1.5–10 cm long, 3 mm in diameter, densely hairy with reflexed hairs; leaf blades broadly ovate to narrowly elliptical, 2.7–14 × 0.6–5.2 cm, apexes acute to obtuse, margins entire, slightly serrulate to sinuate, sometimes densely hairy to glabrous, bases cuneate, sub-cordate, or scutellate-foveate, variously hairy on both surfaces, lateral veins 4–7 pairs, sunken adaxially, densely hairy abaxially. *Inflorescences* 1–5, axillary, cymose, scapiform, flowers 1–3; peduncles 10–18 cm long, 2–3 mm in diameter, sparely hirsute; *bracts* linear to narrowly triangular, 2–6 mm long, ca. 0.5 mm wide at base. *Calyxes* 5-lobed, divided to the base, lobes equal, narrowly triangular to lanceolate-oblong, 8–14 × 0.8–3 mm, sparsely hairy. *Corollas* infundibuliform, 3.5–5 cm long, 1–1.5 cm in diameter at mouth, ca. 0.5 cm in diameter at base, not pouched, sparsely glandular hairy outside, white, violet or white at base and gradually turning into violet to blue towards the lobes, hairiness inside with two yellow strips on lower part of the corolla; corollas distinctly 2-lipped, upper-lips 2-lobed, lobes broadly ovate, 3.5–4 × 7–8 mm, lower-lips 3-lobed, central lobe orbicular, 7–8 × 7–8 mm, lateral lobes broadly ovate, 7–8 × 8–9 mm. *Stamens* 2, filaments 7–32 mm long, adnate to the corolla tube 16–18 mm from the base, slightly curved or geniculate 2 mm from the point of insertion, free part 13–14 mm long and slightly curved, glabrous or apically sparsely glandular hairy; anthers fused by their entire adaxial surfaces, elliptic, ca. 3 mm long, yellowish, thecae divergent. *Staminodes* 3, apex capitate, yellowish, glabrous, 15–21 mm long, adnate to 12–17 mm from the base of the corolla, free part 3.5–5 mm long. Disc a ring, ca. 1 mm high, slightly 5-lobed. *Gynoecium* 2.2–3.5 cm long, 1.4–2 mm in diameter at base, ca. 0.6 mm below the stigma, glabrous or hairy; stigma obscurely 2-lobed, only the lower lip developed, trapeziform, finely hairy. *Capsules* linear, slightly falcate, oblique in relation to the pedicel, reddish brown or greenish and turn to light yellowish, 6–6.5 × 0.4–0.7 cm, glabrous or sparsely hairy on the apical part, dehiscence along the dorsal side; calyx persistent. Seeds long ellipsoid, translucent brownish.

**Etymology.** e new genus *Langbiangia* is named after the Langbiang Plateau. Situated in the southern end of the Annamite Mountain Range and bordered by the Provinces Dak Lak, Khanh Hoa, Lam Dong, and Ninh Thuan (Fig 1), Langbiang Plateau is a biodiversity hotspot [26, 28, 30] and a portion of the plateau within Lam Dong Province was designated by UNESCO as Langbiang Biosphere Reserve in 2015 for its high regional biodiversity that also includes many endangered species [14]. By naming the clade *Langbiangia*, which is thus far only known from the Langbiang Plateau, we are hoping to raise the conservation awareness of this important heritage of biodiversity of Vietnam.

**Distribution.** Vietnam, known only from the Langbiang Plateau (Khanh Hoa and Lam Dong Provinces).

**Habitat.** On fertile soils of forest floors of montane forests.

### Key to the species of *Langbiangia*

1. Leaf blades narrowly elliptical                                      *L*. *poilanei*
1. Leaf blades broadly ovate                                          2

2. Petioles densely pilose; leaf blades flat, with obviously cordate base            *L*. *annamensis*
2. Petioles appressed downward tomentose; leaf blades scutellate             *L*. *scutellifolia*

**1. *Langbiangia annamensis*** (Pellegr.) Luu, C.L.Hsieh & K.F.Chung, **comb. nov.** (Fig 4A) [urn:lsid:ipni.org:names:77306596–1] ≡ *Chirita annamensis* Pellegr. in Fl. Indo-Chine 4: 530 (1930) ≡ *Primulina annamensis* (Pellegr.) Mich.Möller & A.Weber in Taxon 60(3): 781 (2011).
  Type: VIETNAM, Annam, Prov de Nha-trang, massif de Hon-ba, 1000 m, 3 July 1918, *A*. *Chevalier 38697* (lectotype, designated by Wood 1974 [78], p.140: P [MNHN-P-P00602505]).
**2. *Langbiangia poilanei*** (Pellegr.) Luu, C.L.Hsieh & K.F.Chung, **comb. nov.** (Fig 3) [urn:lsid:ipni.org:names:77306597–1] ≡ *Chirita poilanei* Pellegr., Bull. Soc. Bot. France. 73(3): 419. (1926) ≡ *Primulina poilanei* (Pellegr.) Mich.Möller & A.Weber in Taxon 60(3): 784 (2011). (Fig 5).
  Type: VIETNAM, Annam, Giang Ly, Nhatrang dans le lit d’un sui (ruisseau), 2000 m alt. forêt Ouest de Nhatrang, 22 May 1922, *F*. *Poilane 3616* (holotype, P [MNHN-P-P00602520]).
**3 *Langbiangia scutellifolia*** (Luu, N.L.Vu & T.Q.T.Nguyen) Luu, C.L.Hsieh, K.F.Chung, **comb. nov.** (Fig 4B) [urn:lsid:ipni.org:names:77306598–1] ≡ *Primulina scutellifolia* Luu, N.L.Vu & T.Q.T.Nguyen in PhytoKeys 187: 16, *fig 1* (2021).
  Type: VIETNAM, Khanh Hoa Province, Khanh Vinh District, Son Thai Commune, 12°11’39"N, 108°43’30"E, at ca. 1485 m elevation, 01 November 2013, *Luu Hong Truong KH0945* (holotype, SGN!; isotypes SGN!, PHH!, VNMN!).

## Supporting information

S1 Checklist Checklist of inclusivity in global research. (DOCX)

S1 Fig Maximum likelihood phylogeny based on ITS sequences. (PDF)

S2 Fig Maximum likelihood phylogeny based on *trnL-F* sequences. (PDF)

S3 Fig Bayesian inference phylogeny of Didymocarpoideae based on combined ITS and *trnL-F* sequences. (PDF)

S4 Fig A simplified maximum likelihood phylogeny based on combined ITS and *trnL-F* sequences. (PDF)

S5 Fig Maximum likelihood phylogeny based on combined ITS and *trnL-F* sequences. (PDF)

S1 Table NCBI accession numbers of sampled species. (XLSX)

S1 File Nexus file for Bayesian inference. Concatenated alignment of ITS and *trnL-F* sequences and parameters for MrBayes.
(NEX)
