# Supplementary figures and images for: Langbiangia, a new genus of Gesneriaceae endemic to Langbiang Plateau, southern Vietnam and a taxonomic endeavor to achieve key targets of the post-2020 global biodiversity framework
Source: PLoS One. 2023 May 17;18(5):e0284650. doi: 10.1371/journal.pone.0284650 (PMC10191291; doi:10.1371/journal.pone.0284650)

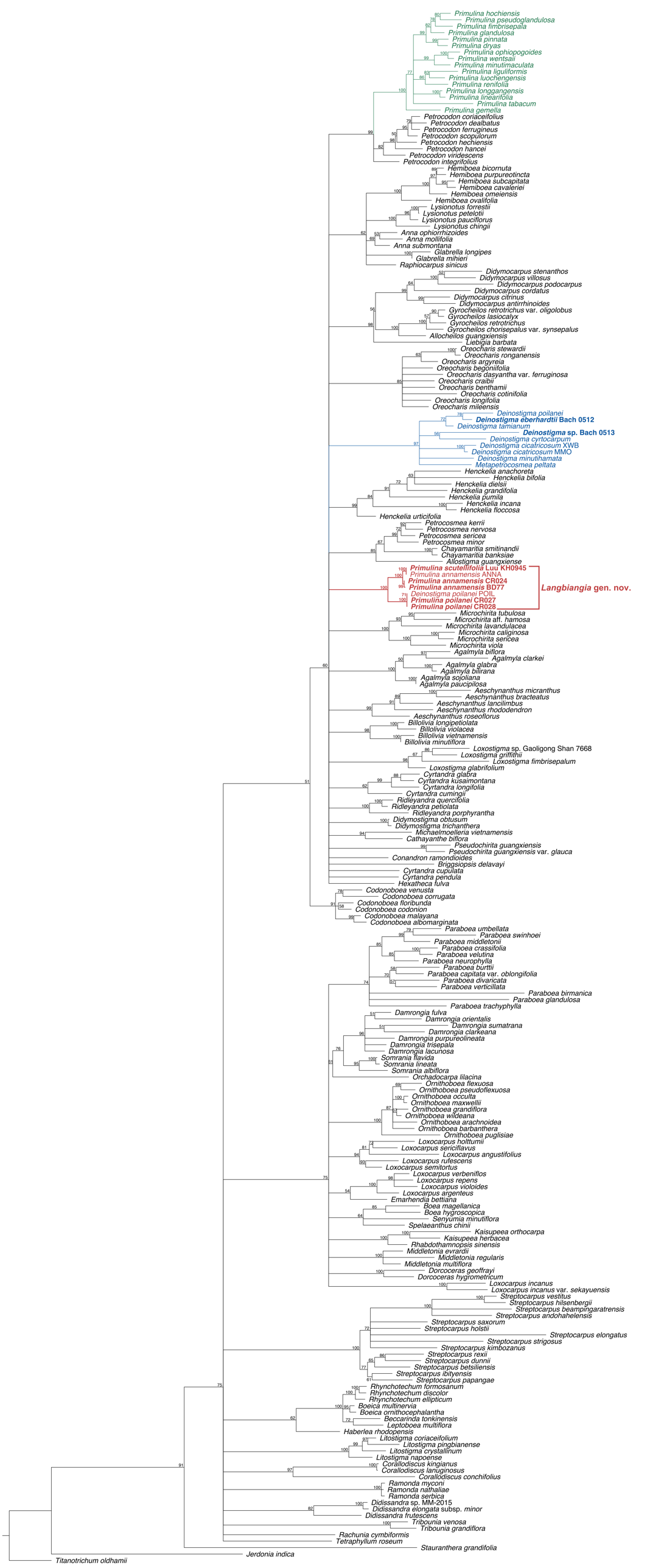

Supplement: S1 Fig — (PDF) [file pone.0284650.s002.pdf]

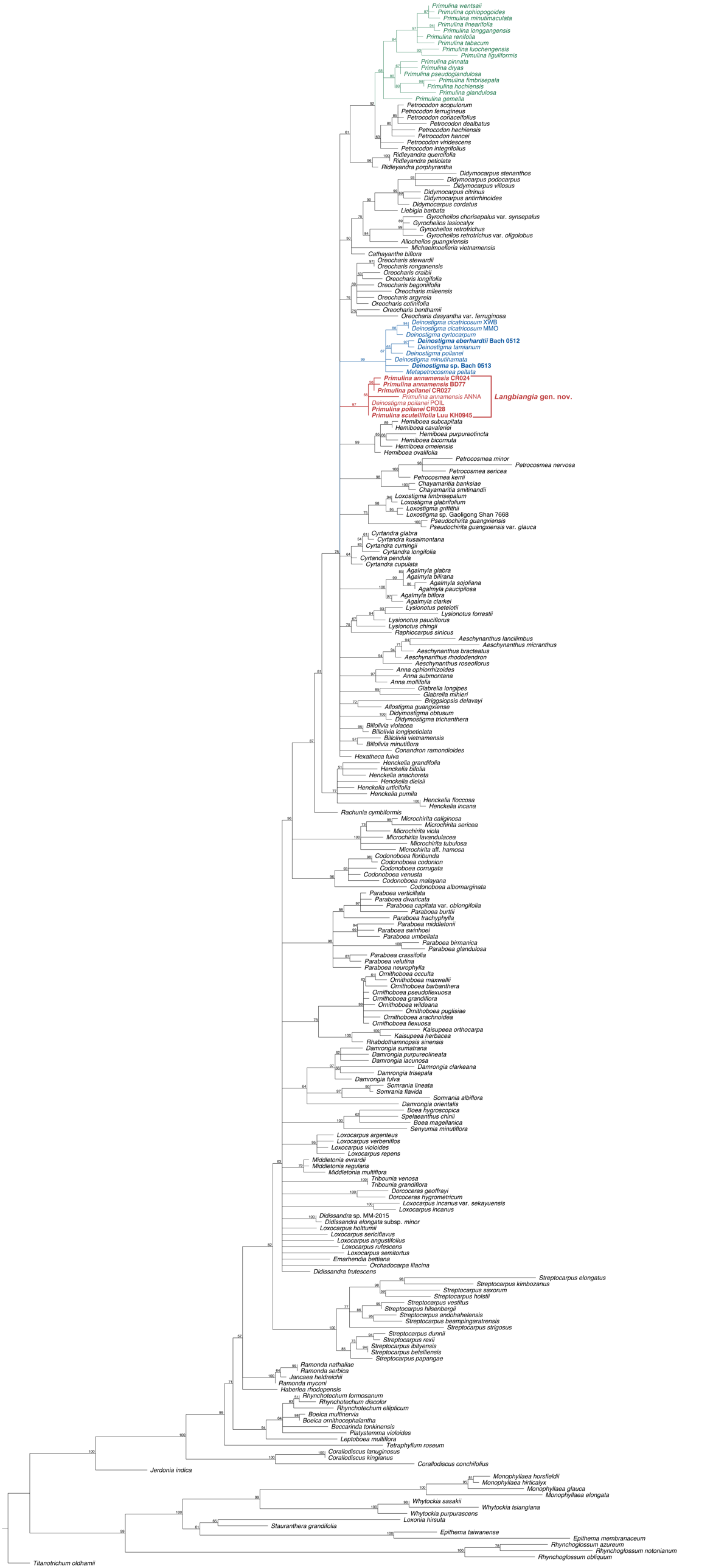

0.01

Supplement: S2 Fig — (PDF) [file pone.0284650.s003.pdf]

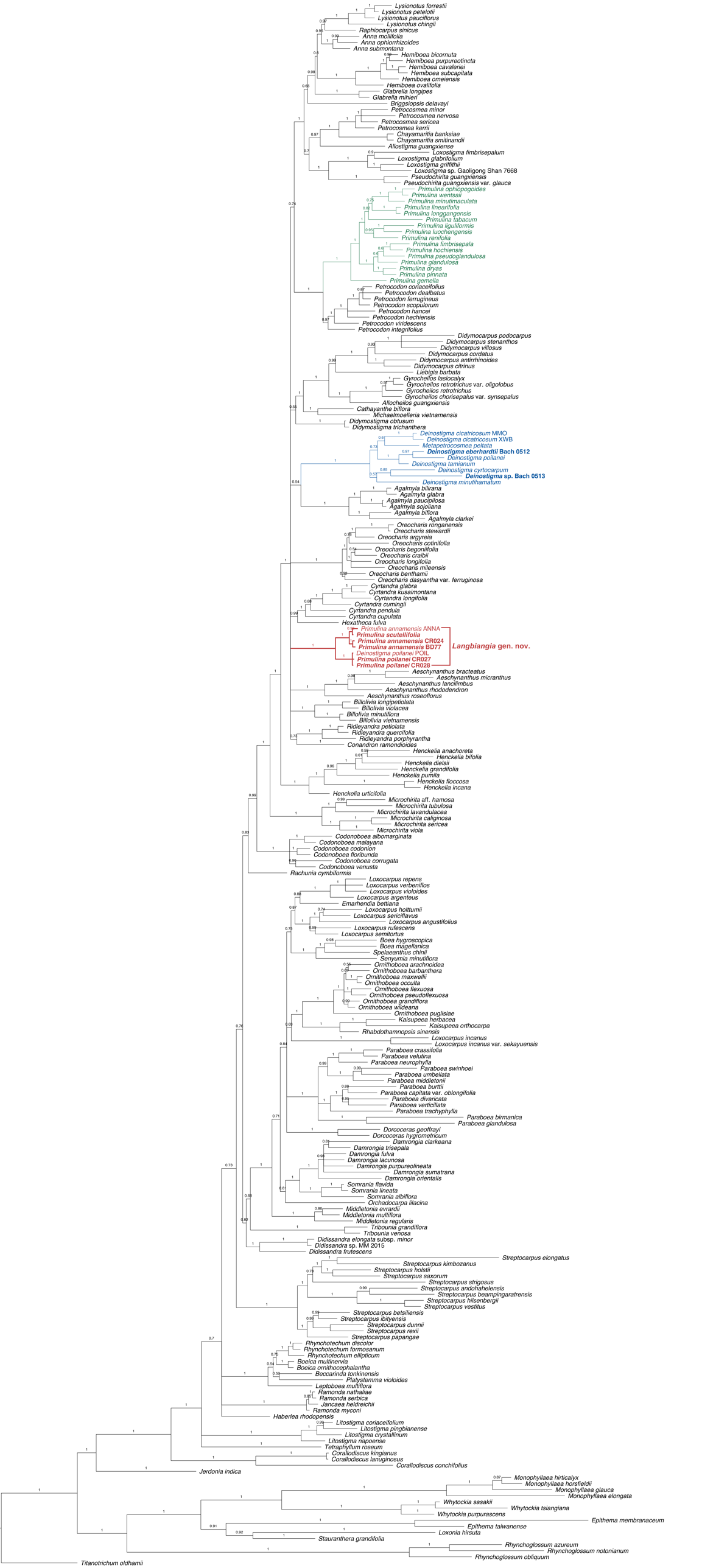

Supplement: S3 Fig — (PDF) [file pone.0284650.s004.pdf]

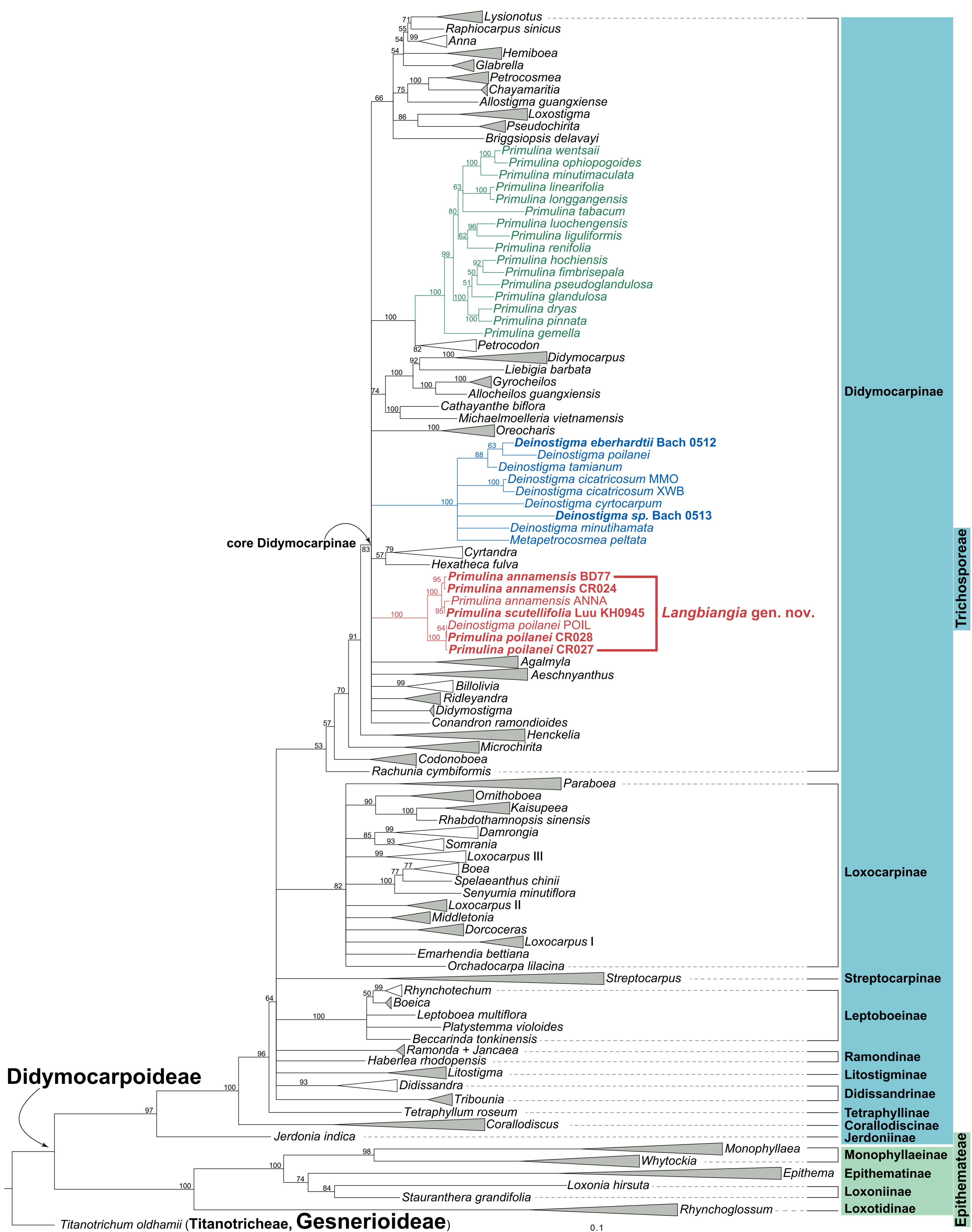

Supplement: S4 Fig — (PDF) [file pone.0284650.s005.pdf]

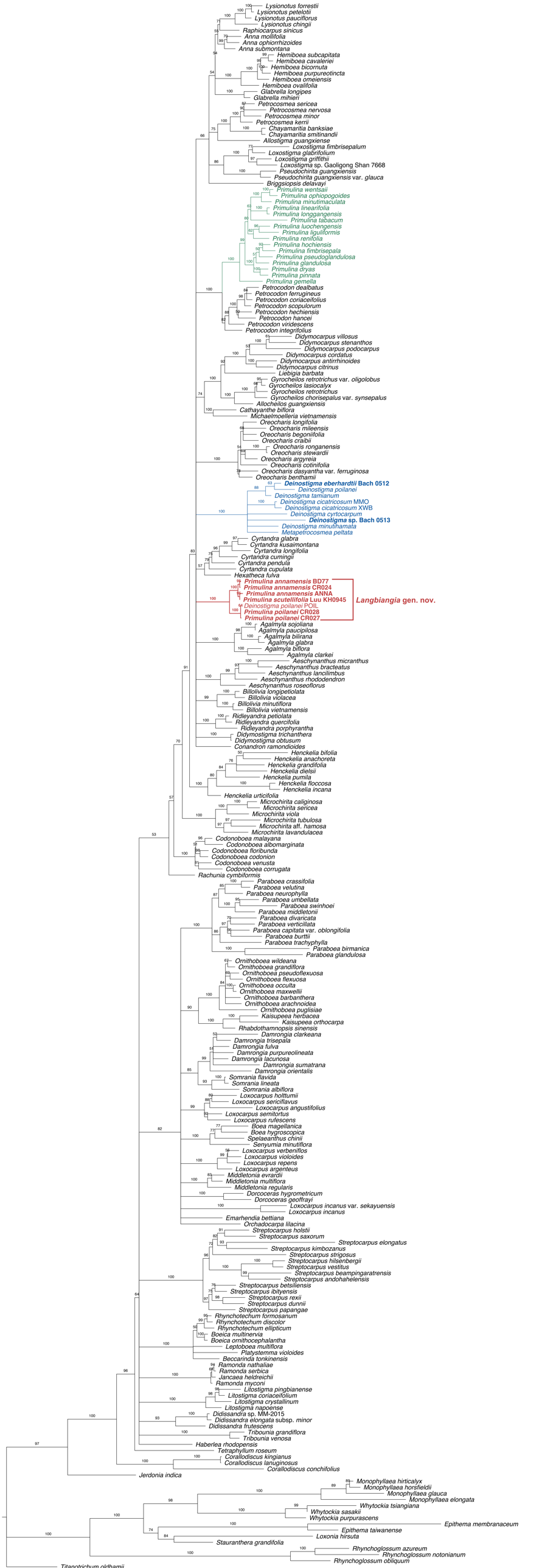

Supplement: S5 Fig — (PDF) [file pone.0284650.s006.pdf]
